# An Instantaneous and Highly Selective Chromofluorogenic Chemodosimeter for Fluoride Anion Detection in Pure Water

**DOI:** 10.1002/open.201300010

**Published:** 2013-04-03

**Authors:** Sameh Elsayed, Alessandro Agostini, Luis E Santos-Figueroa, Ramón Martínez-Máñez, Félix Sancenón

**Affiliations:** [a] Centro de Reconocimiento Molecular y Desarrollo Tecnológico (IDM), Unidad Mixta Universidad Politécnica de Valencia-Universidad de Valencia, Departamento de Química, Universidad Politécnica de Valencia, CIBER de Bioingeniería, Biomateriales y Nanotecnología (CIBER-BNN)Camino de Vera s/n, 46022 Valencia (Spain) E-mail: rmaez@qim.upv.es

**Keywords:** chemodosimeter, colour change, fluoride, hydrolysis, silyl ether

The development of chromofluorogenic sensors for anions is a well-established and studied field in the supramolecular chemistry realm.^1^ This is due to the important roles of anions in biological processes, deleterious effects (such as environmental pollutants), and as toxic compounds and carcinogenic species.^2^

Among inorganic anions, fluoride is widely used in the prevention of dental caries and in osteoporosis treatments.^3^ In spite of these important applications, acute exposure to this anion can cause various diseases, such as nausea, abdominal pain, coma, hypocalcaemia, skeletal fluorosis and osteomalacia.^4^ For all these reasons, the interest in developing chromofluorogenic probes for the selective detection of fluoride has grown in the last few years.^5^

Most of the examples reported for fluoride sensing are based on the use of the “binding site–signalling subunit” approach or on the “chemodosimeter” protocol.^6^ In the probes for which the binding site–signalling subunit approach is used, an optical signalling unit is covalently bonded to a binding site, which usually consists of one H-bond donor moiety or more, such as urea or thiourea groups. The coordination or deprotonation of the acidic protons of the binding sites by fluoride induces changes in colour or fluorescence, which enables anion detection.^7^ In spite of these important features, the chemosensors constructed according to this paradigm present some drawbacks, such as lack of selectivity (other basic anions like cyanide, acetate and dihydrogenphosphate usually give nearly the same optical response) and are unable to display sensing features in competitive media such as water or mixed aqueous environments.

In order to overcome these limitations, the chemodosimeter approach recently has been applied for chromofluorogenic sensing of fluoride anion.5b These probes use selective reactions induced by the target anion, coupled with chromofluorogenic changes. Some of the reported chemodosimeters for fluoride recognition employ this anion’s well-known ability to promote the hydrolysis of silyl ethers.^8^ In particular, several examples of selective chemodosimeters for fluoride anion based on BODIPY derivatives,^9^ terphenyl derivatives,^10^ diphenylacetylenes,^11^ naphthalimides,^12^ benzothiazolium hemicyanine derivatives,^13^ coumarins^14^ and azo dyes,^15^ functionalized with silyl ether moieties, have been recently described. Regardless of the high selectivity of these probes to the fluoride anion, most of them work in organic solvents (acetonitrile, acetone, tetrahydrofuran, dimethyl sulfoxide, dichloromethane) or organic/water mixtures (acetonitrile/water or ethanol/water).

In fact, as far as we know, there are only two molecular-based examples that display sensing features for fluoride in water. One example is based on a *N*-(3-(benzo[d]thiazol-2-yl)-4-(hydroxyphenyl) benzamide derivative that is functionalized with *tert*-butyldiphenylsilyl ether.16a In this case, the chemodosimeter solutions showed an emission band at 418 nm that was progressively quenched and substituted for new fluorescence at 560 nm upon the addition of increasing quantities of fluoride anion. The observed fluorogenic response was ascribed to the hydrolysis of the *tert*-butyldiphenylsilyl moiety, which yielded a highly emissive compound. The second is a very recently published example based on a fluorescein derivative bearing a biocompatible hydrophilic poly(ethylene glycol) polymer and two *tert*-butyldiphenylsilyl ether moieties.16b Aqueous solutions (buffered at pH 7.4) of this chemodosimeter showed a very weak emission band centred at 526 nm (excitation at 490 nm) that increased gradually upon addition of fluoride anion. Again, the observed response was due to the fluoride-induced *tert*-butyldiphenylsilyl ether hydrolysis. Moreover, it is worthy to mention that reported silyl ether-based probes for fluoride detection, showing sensing features in aqueous environments, usually require several minutes (typically in the 3–50 min range) in order to achieve the complete hydrolysis of the silyl ether group.

Bearing in mind the very few examples that the literature reports of the chromo-fluorogenic sensing of fluoride in pure water, and after considering our interest in the development of new optical probes for anions, we designed the novel chemodosimeter **3** for fluoride sensing, based on a pyridine derivative functionalized with a *tert*-butyldimethylsilyl ether group. Compound **3** was able to sense fluoride instantaneously in pure water in the presence of a cationic surfactant. The cationic surfactant was selected as a simple method to solubilize the probe in the inner hydrophobic core of the formed micelles, thus also favouring the inclusion of fluoride anion due to their positively charged shell.

The synthesis of chemodosimeter **3** was achieved by a three-step procedure (Scheme 1). In the first step, 2,6-diphenylpyrylium perchlorate was treated consecutively with methylmagnesium iodide and triphenylcarbenium tetrafluoroborate to give 4-methyl-2,6-diphenylpyrylium tetrafluoroborate (**1**).^17^ Then, compound **1** was condensed with 4-hydroxybenzaldehyde to give stilbene-pyrylium derivative **2**.^18^ Finally, **2** was reacted with ammonium hydroxide (for transformation of the pyrylium ring into a pyridine) and with *tert*-butyldimethylsilyl chloride (TBDMSCl, for transformation of the hydroxyl moiety into a silyl ether), to give the final chemodosimeter **3**. The ^1^H NMR (in CDCl_3_) spectra of **3** showed two singlets centred at 0.25 ppm (6 H) and 1.02 ppm (9 H) ascribed to the methyl and *tert*-butyl groups of the silyl ether group. The most characteristic signals in the aromatic region were one singlet centred at 7.75 ppm, attributed to the two equivalent protons located in the 2,4,6-trisubstituted pyridine heterocycle, and the two doublets with a 15 Hz coupling constant centred at 7.04 and 7.38 ppm, assignable to the hydrogen atoms of the *trans* double bond. HRMS-EI measurements confirmed the structure of the final product with an *m*/*z* value of 464.2403 (464.2331 calculated for C_31_H_34_ONSi^+^).

**Scheme 1:**
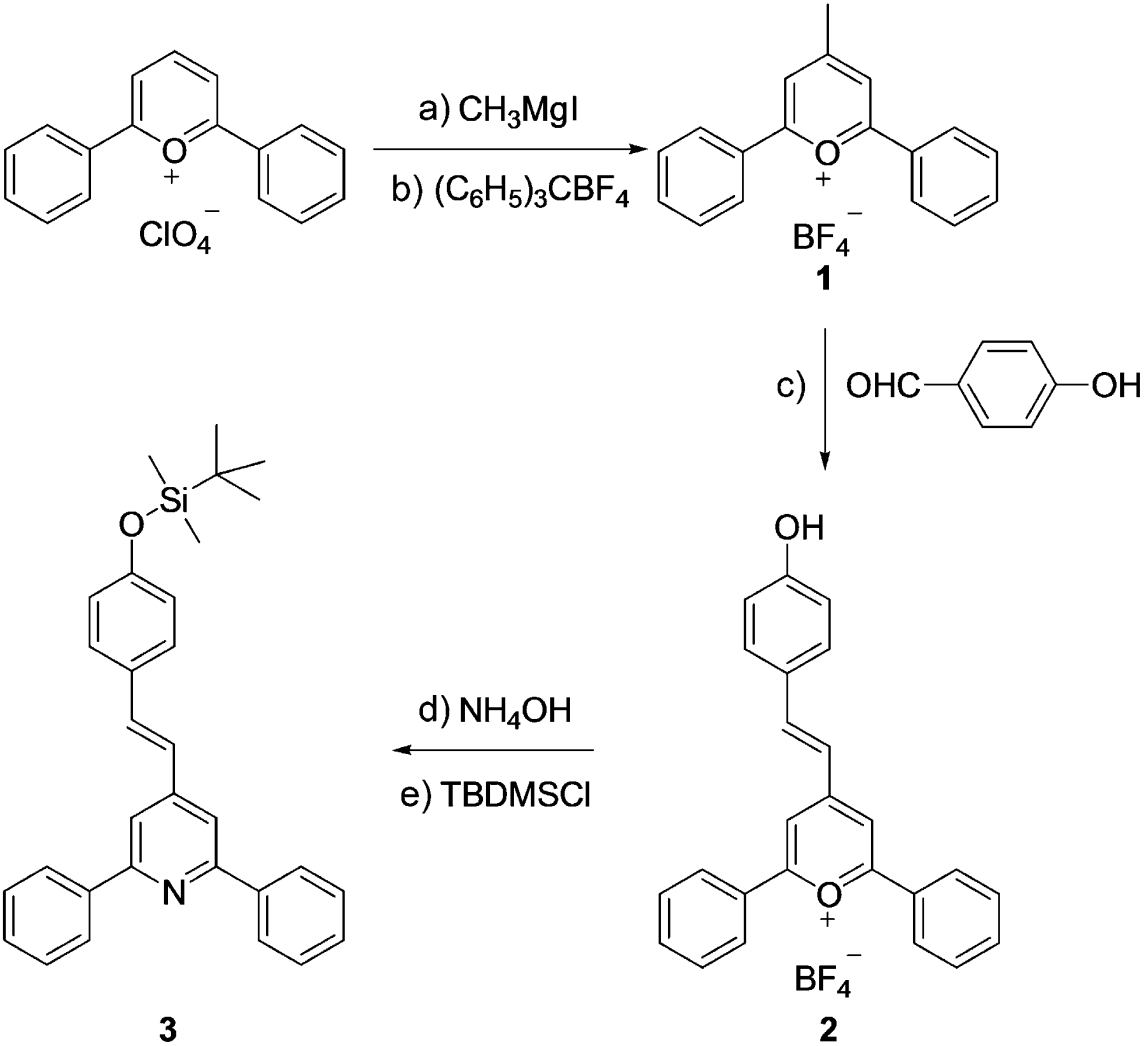
Synthesis of probe **3**. *Reagents and conditions*: a) CH_3_MgI, Et_2_O, RT, 10 h; b) (C_6_H_5_)_3_CBF_4_ (1.06 equiv), RT, 3 h, 66 %; c) 4-Hydroxbenzaldehyde (1.00 equiv), EtOH, reflux, 12 h, 75 %; d) NH_4_OH, RT, 1 h, 93 %; e) TBDMSCl (1.10 equiv), *N*-methylimidazole (3.30 equiv), CH_3_CN, RT, 1 h, 56 %.

As a first step, the chromogenic behaviour of probe **3** in acetonitrile (3.0×10^−5^ mol L^−1^) in the presence of 10 equivalents of selected anions was studied (i.e., F^−^, Cl^−^, Br^−^, I^−^, CN^−^, SCN^−^, AcO^−^, BzO^−^, CO_3_^2−^, NO_3_^−^, ClO_4_^−^, HSO_4_^−^ and H_2_PO_4_^−^). Only the addition of fluoride anion induced remarkable optical changes. In particular, the UV band at 325 nm underwent a hypsochromic shift, while a new band appeared at 445 nm upon fluoride addition (Figure 1). Due to these changes, the solutions of probe **3** changed from colourless to yellow. The other anions tested induced negligible changes in the UV/Vis profile of **3**.

**Figure 1:**
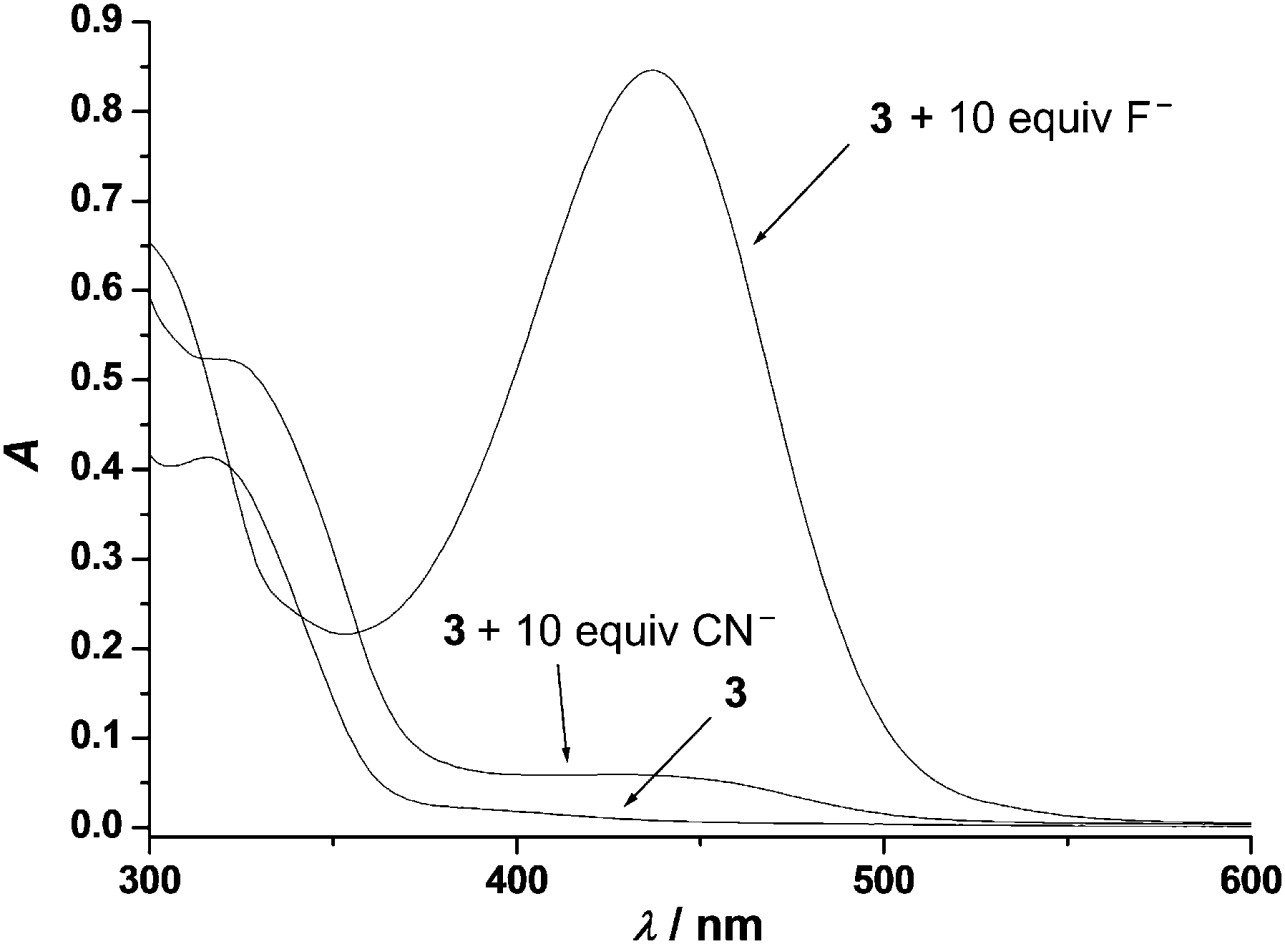
UV/Vis spectra of acetonitrile solutions of chemodosimeter **3** (3.0×10^−5^ mol dm^−3^) in the presence of 10 equivalents of selected anions.

Encouraged by the selective response shown by **3** in acetonitrile, further assays in pure water were performed. UV/Vis studies in the presence of anions were carried out in this highly competitive medium. Probe **3** is weakly soluble in water, but is readily solubilised in water at pH 7.4 containing the cationic surfactant cetyltrimethylammonium bromide (CTABr) at a concentration of 5 mm. The improved solubilisation of **3** in the presence of CTABr is ascribed to the inclusion of **3** into the hydrophobic core of the surfactant micelles. A cationic surfactant was selected to favour the approach of the anionic fluoride to the positively charged CTABr and enhance diffusion of anions into the micelles. The aqueous CTABr solutions of **3** (3.0×10^−5^ mol L^−1^) were also colourless. Moreover, the addition of fluoride anion induced the appearance of a visible band, together with a colour change from colourless to yellow. The other anions tested gave no response (i.e., Cl^−^, Br^−^, I^−^, CN^−^, SCN^−^, AcO^−^, BzO^−^, HCO_3_^−^, NO_3_^−^, ClO_4_^−^, HSO_4_^−^ and H_2_PO_4_^−^).

This fluoride-selective response arises from this known ability of the anion to hydrolyse silyl ether moieties. This hydrolysis induced the formation of a phenolate anion (see structure **4** in Scheme 2) with a strong, negatively charged donor oxygen atom, which is responsible for the appearance of both the red-shifted band at 445 nm and the colour change observed. These results are in agreement with the well-established rule that an increase in the donor ability of a donor moiety in a pull–push chromophore results in a bathochromic shift.^19^ Further studies demonstrated that sensing features of **3** in water containing CTABr and fluoride were observed when the pH is larger than 6.5, whereas lower pH values resulted in no colour changes (data not shown).

**Scheme 2:**
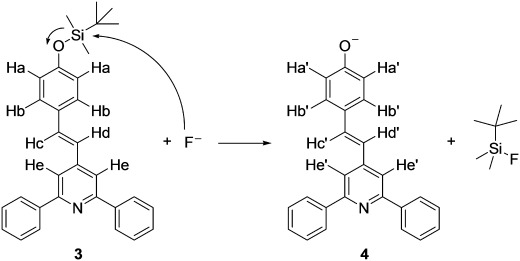
Mechanism of the chromogenic response of **3** in the presence of fluoride anion.

The sensing mechanism was confirmed by ^1^H NMR studies carried out in deuterated acetonitrile. In this solvent, nearly all the signals of **3** underwent upfield shifts in the presence of fluoride (Figure 2; see Scheme 2 for proton assignment). Moreover, the most remarkable shifts were observed for protons He, Ha and Hc. The singlet of the trisubstituted pyridine ring shifted from 7.80 (He) to 7.73 ppm (He′). In addition, the protons of the 1,4-disubstituted benzene rings located in an *ortho* position of the silyl ether moiety shifted from 6.79 (Ha) to 6.37 ppm (Ha′), whereas one of the protons of the double bond shifted from 7.04 (Hc) to 6.71 ppm (Hc′). These upfield shifts are in agreement with the formation of phenolate anion **4** (Scheme 2) by fluoride-induced silyl ether hydrolysis as a key step in the chromogenic response observed.

**Figure 2:**
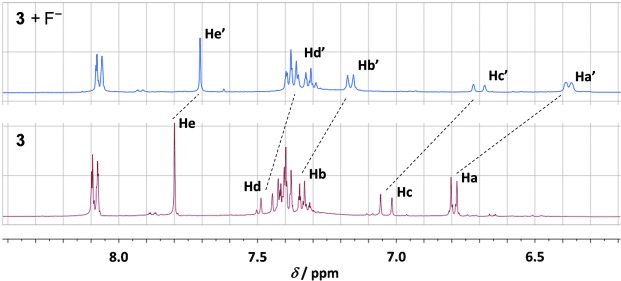
^1^H NMR spectra of probe **3** in CD_3_CN (bottom) and **3** with one equivalent of fluoride anion (top).

Having assessed the selective chromogenic behaviour of probe **3** in water in the presence of anions, fluorogenic studies were also carried out. The solutions of chemodosimeter **3** (3.0×10^−5^ mol L^−1^) at pH 7.4 containing 5 mm CTABr were not fluorescent upon excitation at 445 nm (*Φ*=0.0024). However, the addition of fluoride induced the appearance of an intense emission band centred at 540 nm (*Φ*=0.042), whereas the other anions tested (i.e., Cl^−^, Br^−^, I^−^, CN^−^, SCN^−^, AcO^−^, BzO^−^, HCO_3_^−^, NO_3_^−^, ClO_4_^−^, HSO_4_^−^ and H_2_PO_4_^−^) induced negligible changes in the emission profile of **3** (Figure 3).

**Figure 3:**
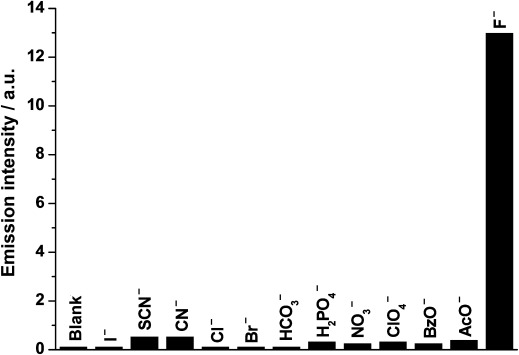
Emission intensity at 540 nm (*λ*_ex_=445 nm) of chemodosimeter **3** (3.0×10^−5^ mol L^−1^) in H_2_O at pH 7.4 containing 5 mm CTABr in the absence (blank) and in the presence of selected anions (10 equiv).

To further characterise the fluorogenic behaviour of chemodosimeter **3**, changes in the emission intensity band at 540 nm were studied in the presence of increasing amounts of the fluoride anion. Figure 4 shows a typical titration profile. From this calibration curve, a detection limit of 1.4 ppm of fluoride was calculated using a conventional fluorimeter. This detection limit is below the recommended value for fluoride content in drinking water (1.8 ppm).

**Figure 4:**
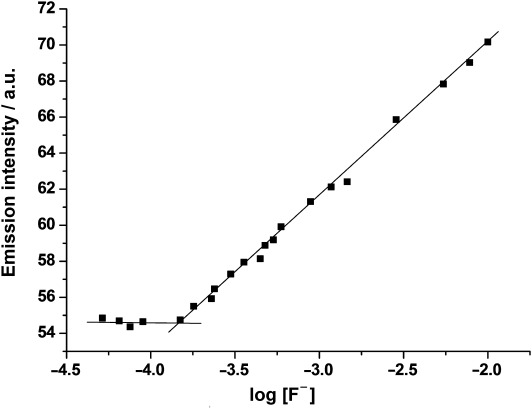
Calibration curve for fluoride using chemodosimeter **3** (3.0×10^−5^ mol L^−1^) in water (pH 7.4) containing 5 mm CTABr.

An additional remarkable feature of probe **3** was the fact that the hydrolysis reaction in the micellar medium was instantaneous. In this sense, it is worth mentioning that other silyl ether-based chemodosimeters for fluoride detection, showing sensing features in aqueous environments (usually mixed organic/water media), usually require several minutes, typically in the range of 3–50 min, to achieve the complete hydrolysis of the silyl ether group, whereas probe **3** displayed changes instantaneously.^14^–^16^

In summary, we report herein the design of a chromo-fluorogenic pyridine-based silyl ether-containing probe for the selective recognition of fluoride anions in water/CTABr solutions. Addition of fluoride anion induces a change in colour from colourless to yellow and the appearance of a strong emission band. Both changes are ascribed to a fluoride-induced hydrolysis of the silyl ether moiety, which yields a phenolate anion. Probe **3** is highly selective and is one of the very few systems for fluoride that displays sensing features in pure water. Moreover, the optical changes are instantaneous, and, as far as we know, our system is the fastest reagent for fluoride signalling in an aqueous environment using silyl ether-based probes.

## Experimental Section

**General**: UV/Vis spectra were recorded with a JASCO V-650 spectrophotometer (Easton, MD, USA). Fluorescence measurements were carried out in a JASCO FP-8500 spectrophotometer. ^1^H and ^13^C NMR spectra were acquired with a 400 MHz Bruker Avance III, whereas mass spectra were carried out with a TripleTOF T5600 spectrometer (AB Sciex, Framingham, MA, USA).

Triethyl orthoformate (98 %), CH_3_I (99 %), *tert*-butyldimethylsilyl chloride (98 %), 4-hydroxybenzaldehyde (98 %), acetophenone (99 %), anhyd CH_3_CN (99.8 %), magnesium, tetraphenylphosphonium tetrafluoroborate and hexadecyltrimethylammonium bromide (CTABr) were purchased from Sigma–Aldrich. Analytical grade solvents, aq NH_4_OH (32 % *w*/*v*), perchloric acid (60 % *v*/*v*) and anhyd MgSO_4_ were purchased from Scharlau (Barcelona, Spain).

**Synthesis of 2,6-diphenylpyrylium perchlorate**: Acetophenone (4 mL, 34.3 mmol) and triethyl orthoformate (10 mL, 60.1 mmol) were placed in a round-bottomed flask (250 mL) under Ar atmosphere at 0 °C. After 15 min of stirring, perchloric acid (60 % *v*/*v*; 7.4 mL, 86.5 mmol) was added dropwise over 30 min. The reaction was allowed to react at RT for 1 h. By addition of Et_2_O (15 mL), the final 2,6-diphenylpyrylium perchlorate derivative was precipitated as a yellow solid (10.1 g, 30.4 mmol, 88.6 % yield). NMR data of the synthesized product agreed with those described in the literature.^20^

**Synthesis of 4-methyl-2,6-diphenylpyrylium tetrafluoroborate (1)**: Magnesium (1 g, 41.1 mmol) was dissolved in anhyd Et_2_O (20 mL), and the resulting solution was added dropwise to a round-bottomed flask containing CH_3_I (5 mL, 80.3 mmol) dissolved in anhyd Et_2_O (30 mL). The mixture was stirred at RT for 1 h, and was added dropwise to a round-bottomed flask containing 2,6-diphenylpyrylium perchlorate (2 g, 8.6 mmol) in anhyd Et_2_O (50 mL) under Ar atmosphere. The crude reaction was stirred at RT for 10 h and poured onto H_2_O (20 mL). After the organic layer was washed with saturated aq NH_4_Cl (2×30 mL) and H_2_O (2×20 mL), dried with anhyd MgSO_4_ and filtered, the solvent was evaporated in vacuo. The sticky residue was dissolved in CH_3_CN (50 mL), and triphenylcarbenium tetrafluoroborate (3 g, 9.1 mmol) was added. The solution was stirred for 3 h at RT. After the solvent was evaporated in vacuo, the residue was dissolved in a minimum volume of acetone, and final product **1** was precipitated with *n*-hexane to give a reddish brown solid (1.9 g, 5.7 mmol, 66 % yield): ^1^H NMR (400 MHz, [D_6_]DMSO): *δ*=2.84 (s, 3 H), 7.78 (t, *J*=6.5 Hz, 4 H), 7.85 (t, *J*=6.5 Hz, 2 H), 8.42 (d, *J*=6.5 Hz, 4 H), 8.83 ppm (s, 2 H).

**Synthesis of 4-((E)-2-(2,6-diphenylpyryliumtetrafluroborate-4-yl)vinyl)phenol (2)**: 4-Hydroxybenzaldehyde (184 mg, 1.5 mmol) was added to a solution of compound **1** (500 mg, 1.5 mmol) in EtOH (50 mL). The reaction was carried out at reflux for 12 h. The solvent was evaporated in vacuo, and the final residue dissolved in a minimum volume of acetone. Precipitation with *n*-hexane gave compound **2** as a reddish solid (463.6 mg, 1.13 mmol, 75 % yield): ^1^H NMR (400 MHz, [D_6_]DMSO): *δ*=7.00 (d, *J*=6.6 Hz, 2 H), 7.45 (d, *J*=15.0 Hz, 1 H), 7.79 (m, 8 H), 8.38 (d, *J*=6.5 Hz, 4 H), 8.67 (d, *J*=15.0 Hz, 1 H), 8.74 ppm (s, 1 H).

**Synthesis of chemodosimeter 3**: Aq NH_4_OH (32 % *v*/*v*; 5 mL) was added to a solution of compound **2** (750 mg, 2.14 mmol) in EtOH (30 mL), and the solution was stirred at RT for 1 h. The solvent was evaporated in vacuo to give a blue solid (700 mg, 2 mmol, 93 % yield). This blue solid (700 mg, 2 mmol) was dissolved in anhyd CH_3_CN (20 mL), and after *tert*-butyldimethylsilyl chloride (424 mg, 2.2 mmol) and *N*-methylimidazole (493 mg, 6.6 mmol) were added, the mixture was stirred at RT for 1 h. The solvent was evaporated in vacuo, and the crude was dissolved in Et_2_O (20 mL), washed with a concd Na_2_S_2_O_3_ (1×30 mL), dried with Na_2_SO_4_, filtered and purified by column chromatography (alumina; hexane/acetone 9:1 *v*/*v*). The chemodosimeter **3** was obtained as a yellowish brown solid (490.6 mg, 1.12 mmol, 56 % yield): ^1^H NMR (CDCl_3_, 400 MHz): *δ*=0.25 (s, 6 H), 1.02 (s, 9 H), 6.89 (d, *J*=6.6 Hz, 2 H), 7.04 (d, *J*=15.0 Hz, 1 H), 7.38 (d, *J*=15.0 Hz, 1 H), 7.48 (m, 8 H), 7.75 (s, 2 H), 8.19 ppm (d, *J*=6.5 Hz, 4 H); ^13^C NMR (100 MHz, CDCl_3_): *δ*=−4.2, 18.4, 25.8, 116.1, 122.9, 128.9, 133.0, 135.0, 135.4, 135.6, 136.3, 141.1, 151.8, 161.2, 176.0, 177.4 ppm; HRMS (EI): *m*/*z* [*M*]^+^ calcd for C_31_H_34_ONSi: 464.2331; found: 464.2403.
